# Dental recommendation and prescribing patterns for systemic analgesics – a cross-sectional study

**DOI:** 10.1007/s00784-025-06403-4

**Published:** 2025-07-14

**Authors:** Diana Heimes, Nina Viktoria Holz, Andreas Pabst, Philipp Becker, Anke Hollinderbäumer, Anita Kloss-Brandstätter, Daniel Müller-Winter, Daniel Stephan, Peer W. Kämmerer

**Affiliations:** 1 Department of Oral and Maxillofacial Surgery, University Medical Center Mainz, Augustusplatz 2, Mainz, 55131 Germany; 2 Department of Oral and Maxillofacial Surgery, Federal Armed Forces Hospital, Rübenacherstraße 170, Koblenz, 56072 Germany; 3 University Medical Center Mainz, Rudolf Frey Lernklinik, Langenbeckstraße 1, Mainz, 55131 Germany; 4 Department of Engineering & IT, Carinthia University of Applied Sciences, Europastraße 4, Villach, 9524 Austria

**Keywords:** Analgesia, Pain management, Preemptive analgesia, Dentist, Tooth extraction, Dental pain

## Abstract

**Objective:**

Pain management is a significant challenge in dental care, making analgesics the most commonly prescribed drugs by dentists. Since many analgesics are available over the counter, data on their use in dental practice is imprecise. This study aimed to gather information on the prescription and recommendation patterns for systemic analgesics among dental practitioners.

**Materials and methods:**

A total of 1,746 dentists were contacted via email and letter and invited to participate in an online survey. The survey covered four sections: personal data, acute and chronic pain management, preemptive analgesia, and managing at-risk patients.

**Results:**

232 dentists participated in the survey. Ibuprofen, typically at 600 mg, was the most commonly recommended analgesic for both acute and chronic pain. For acute pain, analgesics were prescribed for 1–3 days on average. Two-thirds of respondents did not use preemptive analgesia, and of those who did, 96.56% recommended ibuprofen. Notably, in 22.16% of cases, contraindicated analgesics were recommended for patients with renal, liver, or cardiovascular conditions.

**Conclusions:**

The analgesics recommended by dentists are limited in variety and often do not align with specific clinical indications. Nonsteroidal anti-inflammatory drugs, especially ibuprofen, are commonly used long-term, despite conflicting recommendations. A significant number of dentists prescribed contraindicated medications to at-risk patients. Only one-third utilized preemptive analgesia, underscoring the need for improved education and greater use of this approach in dental practice.

**Clinical relevance:**

There is a clear need for enhanced training on analgesic use and preemptive analgesia to improve patient safety, particularly for those with risk factors.

**Graphical Abstract:**

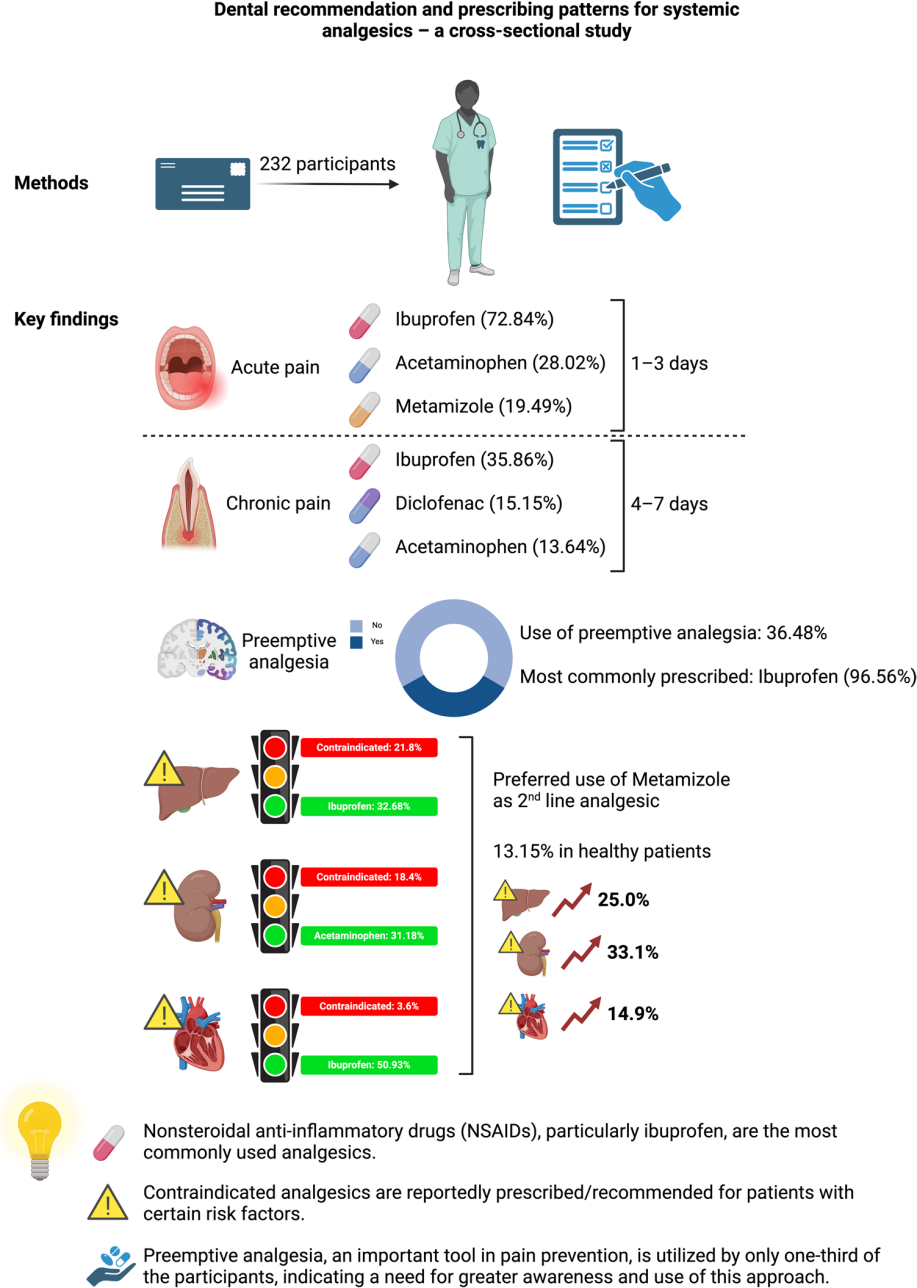

## Introduction

Pain is the most common reason for patients seeking dental emergency care, with most enduring it for more than seven days [1]. The experience of poorly managed pain can lead to patients being challenging to treat, delaying treatment, or even avoiding it altogether [2]. In addition to local anaesthesia, oral systemic analgesics are essential in modern dental therapy. A primary focus in dentistry is the preventive administration of systemically effective analgesics to avoid intra- and post-interventional complications [3], alongside the anti-inflammatory effect of numerous pharmaceuticals [4]. Most common dental procedures, e.g., root canal treatment, periodontal curettage, tooth preparation, and tooth extraction, are typically considered painful and need adequate local and systemic pain management to prevent chronic pain [5]. However, according to the current literature, less than two-thirds of all dentists prescribe analgesics based on the specific diagnosis, and only 30% take the pharmacological properties of the medication into account [6].

The non-evidence-based prescription of medications remains a significant issue in contemporary dental and medical care, resulting in ineffective treatment, adverse effects, and economic burdens on patients and society [6–8]. Common issues include irrational drug combinations, incorrect or unapproved medications for specific purposes, improper therapeutic dosages, and insufficient (too short or too long) therapy durations. These errors often correlate with factors such as the gender and increasing age of the treating dentist [9]. In dental education, significant deficits in prescribing practices have been identified during the student phase [10, 11], which persists into professional practice [12], particularly in the context of analgesic prescription [9, 11, 13]. The non-evidence-based prescription of medications can result in ineffective or even dangerous treatments, harm patient safety, and incur unnecessary costs [14, 15]. For instance, in the US, it was reported that out of 200,000 patients who died from medication-related causes, half succumbed due to overdosing or use of non-indicated drugs [16]. Notably, in Germany, the average person consumes 54 analgesic pills per year, with approximately 1.6 million people dependent on medications (mainly analgesics), though a similarly high number remain undiagnosed [17].

In summary, the various mechanisms of action and the profile of potential side effects ideally require the selection of an analgesic for treating dental pain based on the patient’s medical history, the pharmacological profile of the drug, the intensity and cause of the (anticipated) pain, as well as the cost-to-benefit ratio of the medication. A study demonstrated that more than 3% of dental patients received medications (including NSAIDs) that posed a risk for severe interactions with their continuously used medications [18].

Halling et al. investigated the analgesic prescribing habits of German dentists using health insurance data from 2012–2016 [19]. Due to the availability of many pain medications over the counter (OTC) in Germany, their findings likely underestimate dental analgesic use. Therefore, this study focused not only on prescriptions but also on dentists’ recommendation practices to better reflect real-world analgesic use.

As the dental prescribing and recommendation patterns for analgesics in Germany remain poorly understood, this cross-sectional survey, based on a structured questionnaire, aimed to comprehensively evaluate the types of prescriptions and practitioners’preferences regarding the type, dose, and duration of analgesic therapy.

## Material and methods

### Study design

This study adhered to the CHERRIES (Checklist for Reporting Results of Internet E-Surveys) guidelines. The study was designed as an anonymized cross-sectional cohort study. After approval of the local ethics committee (Nr. 2022–16554; Date: 06/09/2022), all 1,746 private practice dentists across all specialties registered with the Dental Chamber of Rhineland-Palatinate (Germany) were contacted via email and postal mail twice between November 2022 and February 2023. This number represents the total population of private practice dentists registered in Rhineland-Palatinate at the time of the study. Participation was voluntary, anonymous, and based on implied consent after receiving detailed study information. They were invited to participate in an online survey (Table 1) comprising four sections: personal data, acute and chronic pain management, preemptive analgesia, and management of at-risk patients.

**Table 1:** Questionnaire

1. Personal data	
a) Sex	o **Male** o **Female** o **Divers**
b) How many years have you been working as a dentist?	o **Free-text**
c) Do you have a specialization?	o **Yes** o **No**
d) What is your specialization?	o **Orthodontics** o **Parodontics** o **Implantology** o **Prosthodontics** o **Oral surgery** o **Pediatric dentistry** o **Oral and maxillofacial surgery** o **Geriatric Dentistry** o **Endodontology** o **Other: Free-Text**
e) Do you regularly attend professional education events?	o **Yes** o **No**
f) How would you describe the region in which you work?	o **rural** o **urban** o **metropolitan**
2. Acute pain management	
a) Which pain medications do you generally prescribe and recommend when dealing with acute pain?	o **Acetaminophen** o **Ibuprofen** o **Diclofenac** o **Etoricoxib** o **Celecoxib** o **Acetylsalicylic Acid** o **Metamizol** o **Tramadol** o **Tilidine** o **Highly potent opioids (e.g. Oxycodon, Buprenorphin)** o **Other: Free-text**
b) Which two analgesics do you prescribe and recommend most often when dealing with acute pain?	o **Acetaminophen** o **Ibuprofen** o **Diclofenac** o **Etoricoxib** o **Celecoxib** o **Acetylsalicylic Acid** o **Metamizol** o **Tramadol** o **Tilidine** o **Highly potent opioids (e.g. Oxycodon, Buprenorphin)** o **Other: Free-text**
c) Depending on the answer in question 2b: In which dose and frequency do you prescribe *NAME*? E. g In which dose and frequency do you prescribe Acetaminophen?	Typical dosages and frequencies have been given according to the analgesic selected (multiple-choice) E. g o **500 mg** o **1000 mg** o **1–2 times per day** o **3–4 times per day** o **5–6 times per day** o > **6 times per day**
d) For how long do you typically prescribe or recommend analgesics when dealing with acute pain such as pulpitis, abscess, or after invasive surgical procedures?	o > **4 weeks** o **2–4 weeks** o **8–14 days** o **4–7 days** o **1–3 days**
e) What would be your analgesic procedure after wisdom tooth osteotomy? Please list product, dosage, frequency and duration of use	o **Free-text**
f) Please list the factors that affect your choice of recommended/prescribed analgesic. Which medical anamnestic factors cause you to consider adjusting your standard analgesic regimen?	o **Free-text**
3. Chronic pain management	
a) Approximately how many patients per year do you treat suffering from chronic pain (> 3 months)?	o **Free-text**
b) Which pain medications do you generally prescribe and recommend when dealing with chronic pain?	o **Acetaminophen** o **Ibuprofen** o **Diclofenac** o **Etoricoxib** o **Celecoxib** o **Acetylsalicylic Acid** o **Metamizol** o **Tramadol** o **Tilidine** o **Highly potent opioids (e.g. Oxycodon, Buprenorphin)** o **Tricyclic antidepressants (amitriptyline, doxepin, clomipramine, imipramine)** o **Anticonvulsants (carbamazepine, gabapentin, pregabalin)** o **Other: Free-text**
c) Which two analgesics do you prescribe and recommend most often when dealing with chronic pain?	o **Acetaminophen** o **Ibuprofen** o **Diclofenac** o **Etoricoxib** o **Celecoxib** o **Acetylsalicylic Acid** o **Metamizol** o **Tramadol** o **Tilidine** o **Highly potent opioids (e.g. Oxycodon, Buprenorphin)** o **Tricyclic antidepressants (amitriptyline, doxepin, clomipramine, imipramine)** o **Anticonvulsants (carbamazepine, gabapentin, pregabalin)** o **Other: Free-text**
d) For how long do you typically prescribe or recommend analgesics when dealing with chronic pain such as chronic toothache or neuropathic pain?	o > **4 weeks** o **2–4 weeks** o **8–14 days** o **4–7 days** o **1–3 days**
e) What would be your analgesic procedure for a patient presenting unsuccessful root canal treatment and subsequent tooth extraction with persistent pain for several months on ibuprofen, acetaminophen and metamizole? Please specify product, dosage, frequency, and duration of use	o **Free-text**
3. Preventive/Preemptive Analgesia	
a) Do you use preventive/preemptive analgesia, i.e. analgesic administration before the scheduled procedure (on the same day)?	o **Yes** o **No**
b) What analgesic do you prescribe or recommend to patients for preventive/preemptive analgesia?	o **Acetaminophen** o **Ibuprofen** o **Diclofenac** o **Etoricoxib** o **Celecoxib** o **Acetylsalicylic Acid** o **Metamizol**
4. Patient factors	
a) Which analgesics would you choose to treat acute postsurgical symptoms (e.g., after wisdom tooth removal) in a patient with liver dysfunction?	o **Acetaminophen** o **Ibuprofen** o **Diclofenac** o **Etoricoxib** o **Celecoxib** o **Acetylsalicylic Acid** o **Metamizol** o **Tramadol** o **Tilidine** o **Highly potent opioids (e.g. Oxycodon, Buprenorphin)**
b) Which analgesics would you choose to treat acute postsurgical symptoms (e.g., after wisdom tooth removal) in a patient with renal dysfunction?	o **Acetaminophen** o **Ibuprofen** o **Diclofenac** o **Etoricoxib** o **Celecoxib** o **Acetylsalicylic Acid** o **Metamizol** o **Tramadol** o **Tilidine** o **Highly potent opioids (e.g. Oxycodon, Buprenorphin)**
c) Which analgesics would you choose to treat acute postsurgical symptoms (e.g., after wisdom tooth removal) in a patient with cardiovascular disease)	o **Acetaminophen** o **Ibuprofen** o **Diclofenac** o **Etoricoxib** o **Celecoxib** o **Acetylsalicylic Acid** o **Metamizol** o **Tramadol** o **Tilidine** o **Highly potent opioids (e.g. Oxycodon, Buprenorphin)**

### Questionnaire

To ensure comparability of results and minimize bias, response options were provided either dichotomously (yes/no) or as multiple- and single-choice questions, with the inclusion of free-text fields. The inclusion of free-text options was designed to prevent bias by allowing participants to provide responses that better reflected their prescribing practices, rather than being confined to predetermined multiple-choice answers crafted by the authors. Furthermore, no dynamic questions were used; each question was presented independently, requiring individual responses. This design ensured that each response could be uniquely analyzed without the influence of subsequent or related questions.

The questionnaire was specifically designed for this study and included 17 closed and 5 open-ended questions, each individually analyzable. Initial review was performed internally through careful test runs and in-depth discussions. External validation was then carried out with three experienced board-certified OMFS surgeons and three OMFS residents from various departments, none of whom were involved in the study itself. Discrepancies or issues identified during the review process were addressed through collaborative discussions, and the questionnaire was refined iteratively. While an extensive internal review and external validation by a panel of experienced board-certified oral and maxillofacial surgeons and residents were performed to ensure face and content validity, a formal test–retest reliability assessment was not conducted. The survey began by gathering general demographic information, including participants’ gender, age, years of experience, and specialty. Respondents were also asked about their regular attendance at postgraduate educational events and whether they worked in an urban, rural, or metropolitan setting.

The second section focused on analyzing prescription and recommendation modalities. First, the participants were asked which analgesic they prescribe/recommend for patients with acute dental pain, followed by a second question on which medication they use most frequently. Furthermore, the participants were required to state the dosage and frequency of the prescribed/recommended medication. This section concluded with questions about the analgesic regimen of a wisdom tooth extraction and factors influencing the choice of drugs.

The third section addressed the prescription and recommendation modalities for chronic pain management. The participants needed to state which analgesic they use in such cases and which ones they prescribe/recommend most often. The final question aimed to gather information on the analgesic regimen in the case of a patient with chronic dental pain who had already been treated with non-opioid analgesics for several weeks.

The fourth section was meant to analyze whether the participants use preemptive analgesia and, if so, which medication they prefer.

The final section required participants to state how they would treat patients with certain risk factors (liver, renal, and cardiovascular disease) in case of acute dental pain.

### Survey administration and response rate

The survey was administered online via LimeSurvey (LimeSurvey GmbH, Hamburg, Germany). Out of the 1,746 invited dentists, 232 completed the survey, resulting in a response and completion rate of 13.29%.

### Data protection

All data were collected anonymously. No personal identifiers were recorded, and participant confidentiality was maintained throughout the study.

### Statistics

The survey was created using LimeSurvey (LimeSurvey GmbH, Hamburg, Germany), and the collected data were automatically exported to Microsoft Excel (Microsoft Corporation, Redmond, WA, USA) for analysis. Quantitative analyses were performed for all questionnaire items. Percentages were calculated based on the total number of participants who completed the survey. Since not all participants answered every single question, some percentages may not add up to exactly 100%. In questions allowing multiple responses, percentages may exceed 100%, as each selected option was calculated relative to the total number of survey participants rather than the number of responses per item. This approach was chosen to ensure consistency and comparability across all reported data. Free-text responses were manually reviewed, categorized, and quantitatively summarized as percentages. Descriptive statistics were used to present the distribution of responses across the sample, facilitating an intuitive interpretation of the findings in relation to the study objectives. All statistical graphs were generated using Microsoft Excel.

## Results

### Demographics/Personal data

A total of 232 dentists (= 100%) completed the questionnaire, with 59.91% identifying as male, 28.45% as female, and 0.86% as divers. The participants’ years of experience in dentistry ranged from 1 to 49 years (Mean [M] = 23.58 ± 11.34 years). 17.96% of participants had 1–10 years of work experience, 22.33% worked for 11–20 years (30.58% for 21–30 years, and 29.12% for over 30 years). Due to the anonymized evaluation, it was not possible to match the questionnaire responses with the demographic data of the participants.

A total of 27.59% of the respondents reported having a speciality, with most specializing in dental implantology, followed by periodontology, oral surgery, and endodontology. The majority of participating dentists (82.76%) reported attending professional education events regularly. Regarding practice location, only 7.33% reported working in a metropolitan area, while 30.60% practiced in urban and 51.29% in a rural area.

### Management of acute pain

**Fig. 1:**
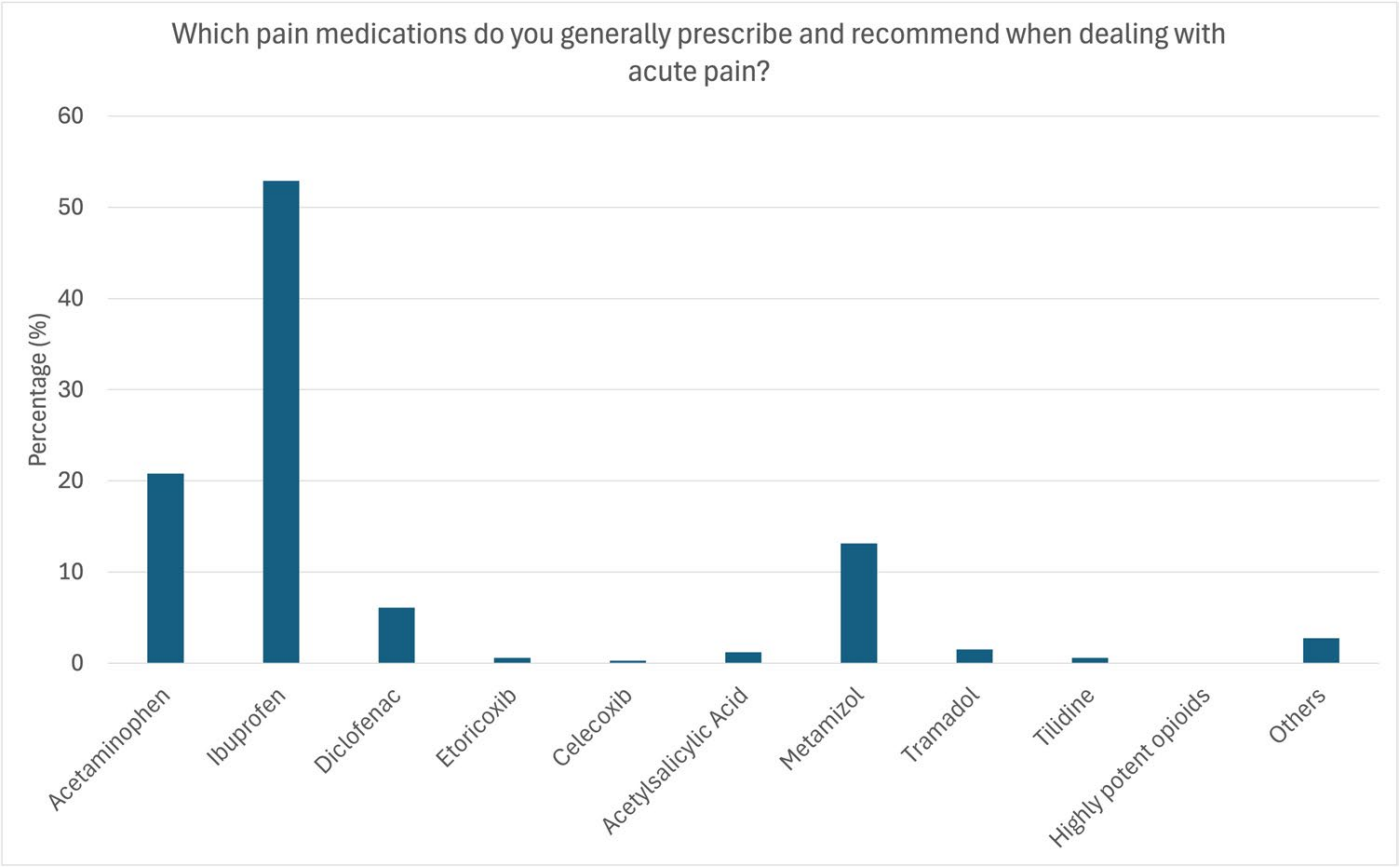
Analgesics prescribed for the treatment of acute dental pain. Ibuprofen was reportedly prescribed/recommended most often for the treatment of acute dental pain (52.9%), followed by acetaminophen (20.8%), metamizole (13.1%), and diclofenac (6.1%)

Ibuprofen was reported to be prescribed and recommended most often in dental practice (72.84%), followed by acetaminophen (28.02%), metamizole (19.49%), and diclofenac (7.76%). Only five participants reported prescribing tilidine as an analgesic, three prescribed acetylsalicylic acid, and two used either etoricoxib or tramadol. Notably, highly potent opioids were not reported to be prescribed by dentists (Fig. 1).

Acetaminophen is primarily prescribed in 500 mg doses (80.6%), with the majority recommending it three to four times daily (63.16%). Only 13 participants reported using a 1000 mg dosage of acetaminophen, with six dentists recommending it five to six times daily.

Ibuprofen was reportedly used in doses of 600 mg (67.01%), followed by 400 mg (20.42%) and 800 mg (12.57%). Participants predominantly recommended a 400 mg dose on an as-needed basis, followed by administration three to four times daily. For the 600 mg dose, the most common regimen was three times daily, followed by four to five times daily; only a few participants recommended it on an as-needed basis or one to two times daily. The 800 mg dose was most frequently recommended on an as-needed basis, followed by three to four times and two to three times daily. Additionally, one participant reported recommending a 1200 mg dose to be taken twice daily.

For diclofenac, most dentists prescribed a dose of 50 mg (76.92%) rather than 25 mg, both being recommended one to two times daily (86.67%). Etoricoxib was reportedly used in doses ranging from 60 to 90 mg, one to two times per day. The participants did not provide answers regarding daily doses and frequencies of celecoxib, metamizole, or acetylsalicylic acid.

**Fig. 2:**
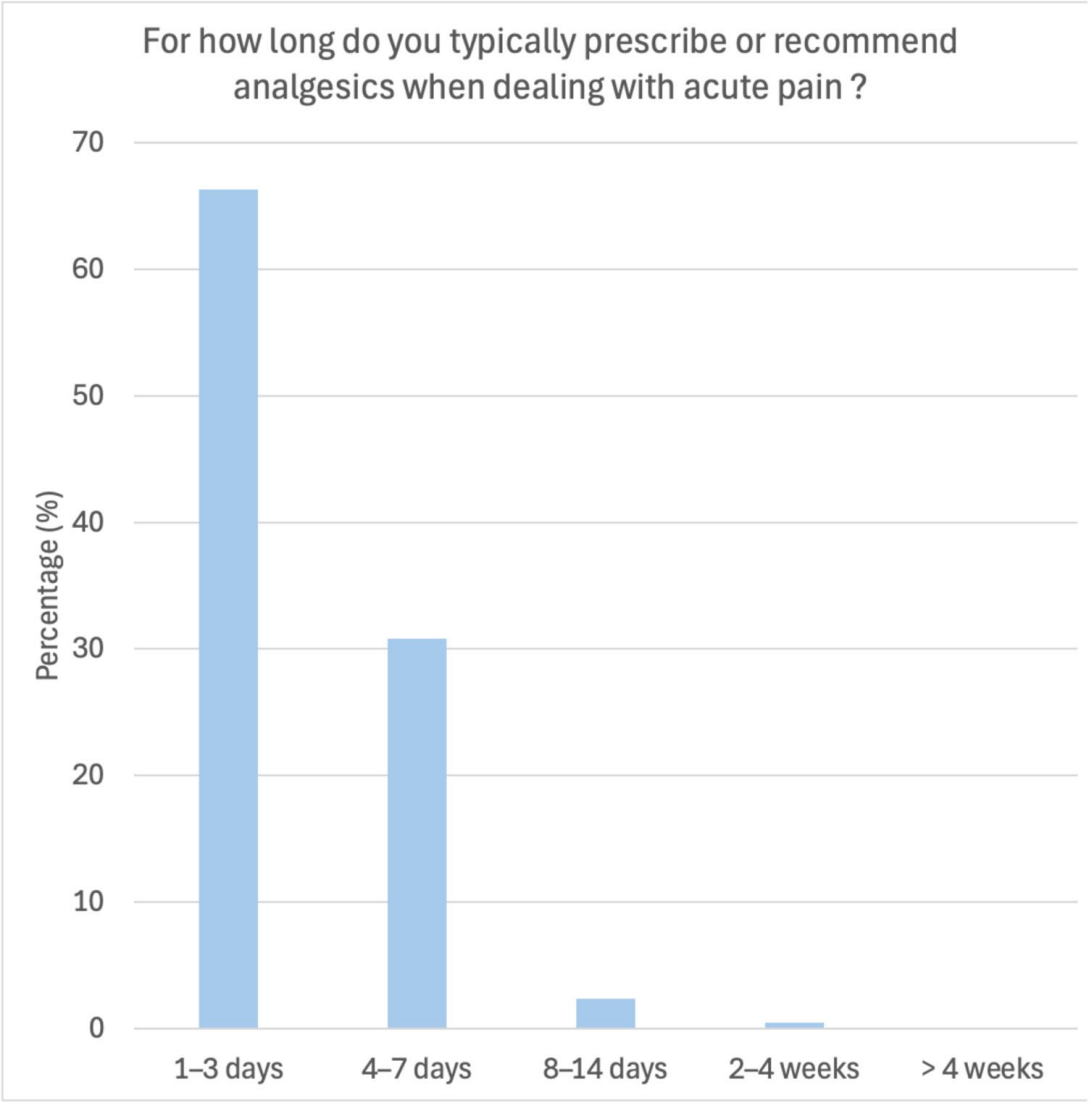
Duration of analgesic therapy for acute pain treatment. Analgesics were reportedly prescribed/recommended most often for short-term intervals (1–7 days) (97.1%)

When asked for the treatment duration of patients suffering from acute pain like pulpitis, abscess, or after invasive surgical procedures, 66.67% reported recommending analgesics for one to three days, 31.88% for four to seven days, and only three dentists suggested extending the treatment beyond this period (Fig. 2).

The participants were asked about their analgesic protocols following wisdom tooth osteotomy. A total of 87.36% of the participants recommend/prescribe ibuprofen, with 40.79% of those suggesting 600 mg as needed, 7.89% recommending 800 mg, and 11.84% advising 400 mg. In 36.84% of cases, participants recommend ibuprofen intake according to a particular protocol. Regular intake was recommended for 1–14 days (1–2 days: 30.19%; 3–5 days: 39.62%; 5–7 days: 28.30%; 10–14 days: 1.89%). Although the maximum daily dose of ibuprofen is 2400 mg according to the medical information, doses exceeding this limit were recommended in four cases. In 9 out of 174 cases, dentists recommend combinations of different analgesics (ibuprofen and metamizole/acetaminophen/cortisone). Metamizole was prescribed in 5.75% of cases, primarily on an as-needed basis, and acetaminophen in 2.87% of cases. Rarely recommended/prescribed analgesics included naproxen, etoricoxib (both cases with dramatic overdosages of 180–250 mg/day), diclofenac, and codeine (up to six times daily). Only one dentist would not recommend/prescribe any medication.

When asked about the factors influencing the dentist’s choice of recommended/prescribed analgesia, 17.14% of participants named allergies as determining factor, followed by renal diseases (10.86%), the patient’s opinion/whish (9.14%), liver diseases (8.29%), indication (7.43%), gastrointestinal diseases (7.43%), comedication (7.14%), general diseases (6.86%), age (5.12%), intake of anticoagulants (4.86%), pregnancy (3.71%), cardiovascular diseases (3.14%). Less frequently mentioned factors included body weight, intake of other pain medications, asthma, addiction, smoking, costs, pain medication abuse, and ethnicity. Three participants stated that they did not adapt to the patient’s characteristics.

### Management of chronic pain

The participants stated treating an average of 7.72 (± 25.43; Min. = 0; Max. = 250) patients with chronic pain per year on average.

When asked which analgesics dentists prescribe/recommend for patients with chronic pain in general, ibuprofen was chosen in 28.57% of cases, followed by diclofenac in 16.67%. Metamizole was used in 11.43%, acetaminophen in 8.57%, while selective COX-2 inhibitors were prescribed less frequently (etoricoxib 0.95%, celecoxib 0.48%). Only 4.77% reported prescribing low-potency opioids like tramadol and tilidine, and 1.43% of participants prescribed high-potency opioids like oxycodone or buprenorphine. Tricyclic antidepressants like amitriptyline, doxepin, clomipramine, and imipramine were used in 2.86% of cases, and antipsychotic agents like carbamazepine, gabapentin, and pregabalin in 4.76%. Additionally, 18.57% would also recommend physiotherapy, NSAIDs other than those mentioned above (oxaceprol), acupuncture, and referral to an expert (5.7%).

**Fig. 3:**
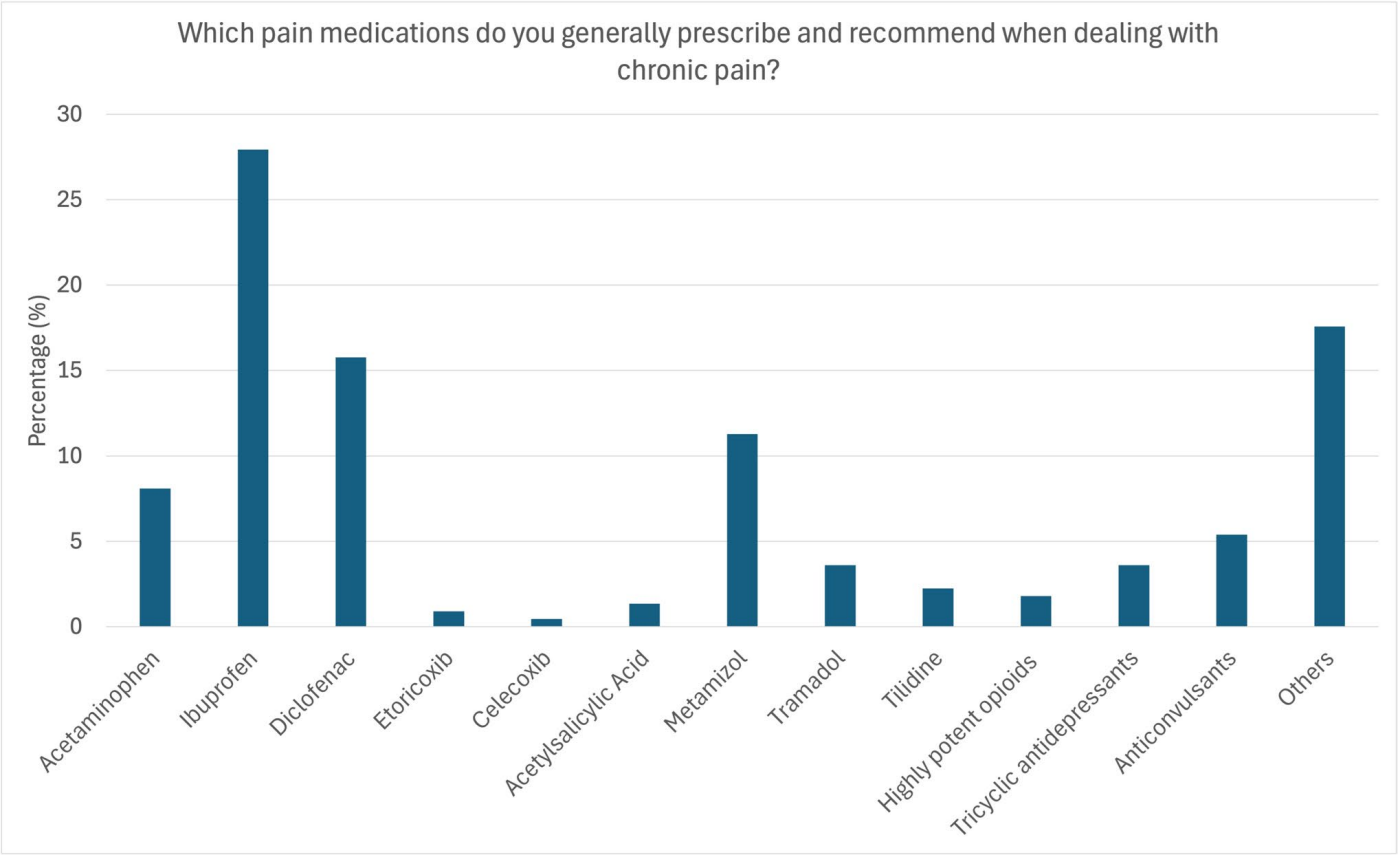
Analgesics prescribed for the treatment of chronic dental pain. Ibuprofen was reportedly prescribed/recommended most often for the treatment of acute dental pain (27.9%), followed by diclofenac (15.8%), metamizole (11.3%), and acetaminophen (8.1%). Co-analgesics like tricyclic antidepressants and anticonvulsants were only prescribed in only 10% of cases

In terms of most frequently prescribed analgesics, the dentists reported using ibuprofen (35.86%) most often, followed by diclofenac (15.15%), acetaminophen (13.64%), and metamizole (9.6%). Other analgesics were used significantly less frequently (Fig. 3).

**Fig. 4:**
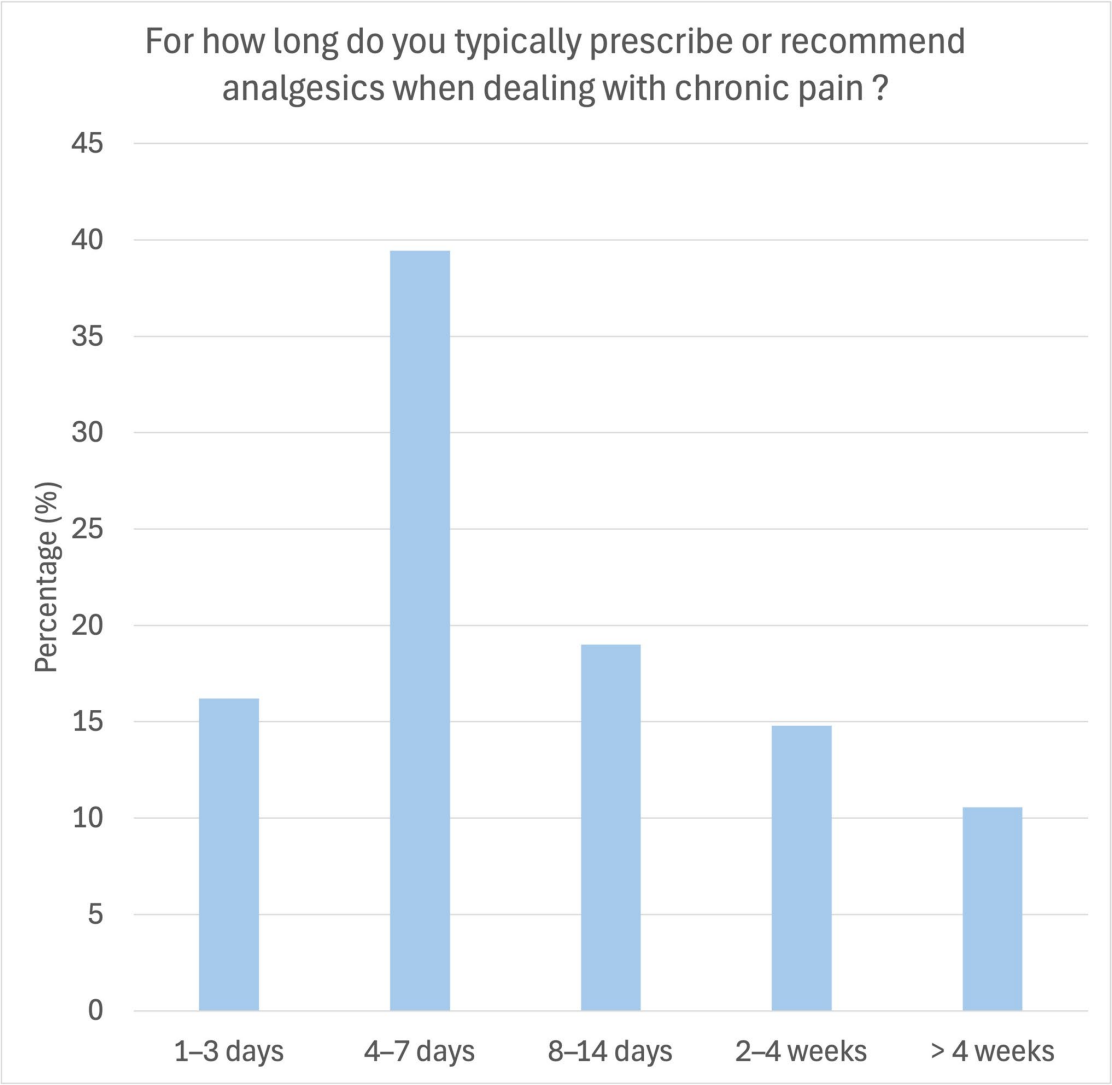
Duration of analgesic therapy for chronic pain treatment. Analgesics were reportedly prescribed/recommended most often for a short period of time (4–7 days; 39.4%). In 19% of cases, the participants reported to prescribe/recommend analgesia intake for up to 14 days, 14.8% for 2–4 weeks and 10.6% for longer than 4 weeks

When asked about the duration of treatment for patients with chronic pain, 40.60% of participants reported treating for 4–7 days, 18.8% for 8–14 days, 17.29% for only 1–3 days, 13.53% for 2–4 weeks, and 9.77% for longer than four weeks (Fig. 4).

The participants were further asked about their analgesic procedure for a patient with persistent pain following unsuccessful root canal treatment and subsequent tooth extraction despite treatment with ibuprofen, acetaminophen, and metamizole for several months. One-fifth of the participants stated they would not treat patients who experience pain for longer than a few days (21.88%), choosing instead to refer the patient to an expert (20.83%). Eight dentists stated not to change the current treatment strategy, whereas 16.67% would recommend taking 400–800 mg of ibuprofen multiple times a day ranging from on-demand use to continuous treatment over several weeks. Metamizole and diclofenac were reportedly recommended in 9.38% of cases. Some dentists suggested using prednisolone or antibiotics instead of analgesics. Low-potency opioids were reported to be used in 6.25% of cases and antipsychotic agents in 5.21%. Wound revision in combination with an antibiotic agent would be performed in 7.3%.

### Preemptive analgesia

The participants were asked whether they use preemptive analgesia in their dental practice. One-third stated using this approach, whereas the majority (63.52%) did not employ preemptive analgesia.

**Fig. 5:**
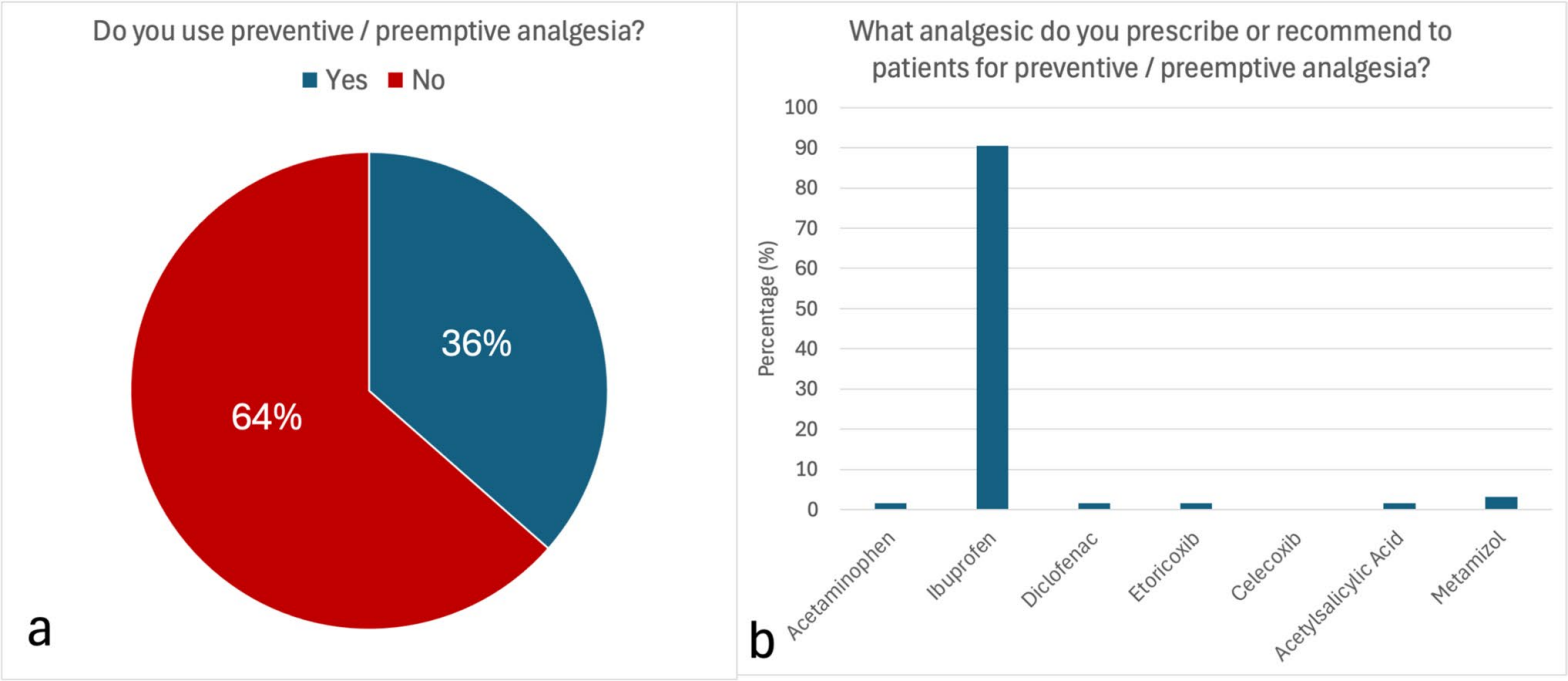
**a** Percentage of participants using preemptive analgesia to prevent pain during and after surgery. **b** Analgesics prescribed for the preemptive analgesia. Here, ibuprofen was reportedly prescribed/recommended most often (90.5%)

Among those who did, nearly all prescribed ibuprofen for preemptive analgesia (96.56%) (Fig. 5).

### Analgesia in patients at risk

**Fig. 6:**
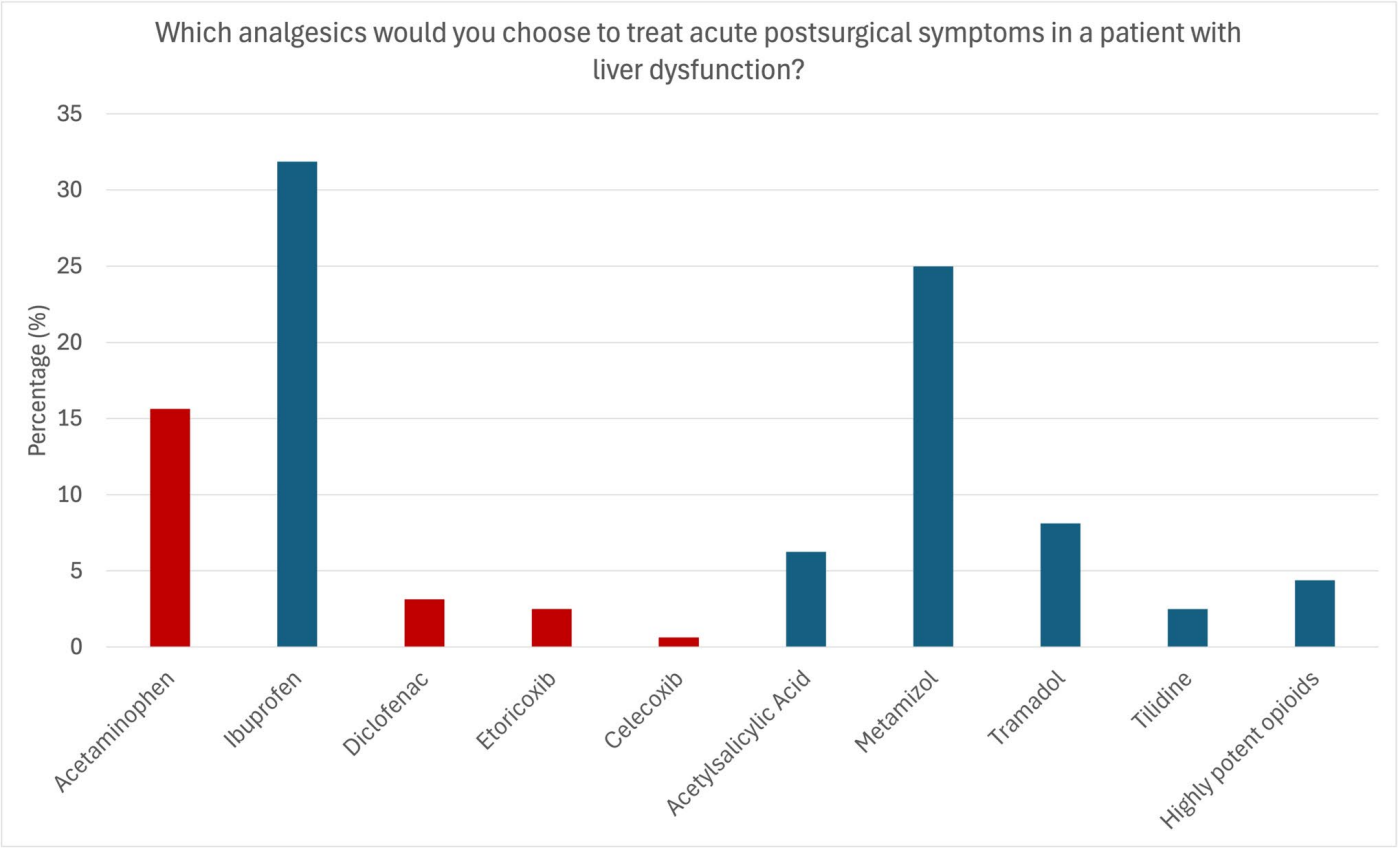
Analgesics preferably prescribed to treat acute pain in patients with liver dysfunction. The participants reported to preferably prescribe/recommend ibuprofen (31.9%) and metamizole (25%). Contraindicated analgesics were used in 21.8% (acetaminophen: 15.6%, diclofenac: 3.1%, etoricoxib: 2.5%, celecoxib: 0.6%) and highly potent opioids in 4.4%. Red: Contraindicated medication

The participants were asked which analgesics they prescribe/recommend for treating acute postsurgical symptoms (e.g., after wisdom tooth removal) in a patient with liver dysfunction. Most dentists would prescribe/recommend ibuprofen (32.68%), followed by metamizole (24.84%) and acetaminophen (15.03%). Tramadol was chosen in 7.84% of cases, acetylsalicylic acid in 6.54%, high-potency opioids in 4.58%, and diclofenac in 3.27%. Notably, contraindicated medications were reportedly prescribed/recommended in 27.45% of cases (acetaminophen, diclofenac, selective COX-2 inhibitors) (Fig. 6).

**Fig. 7:**
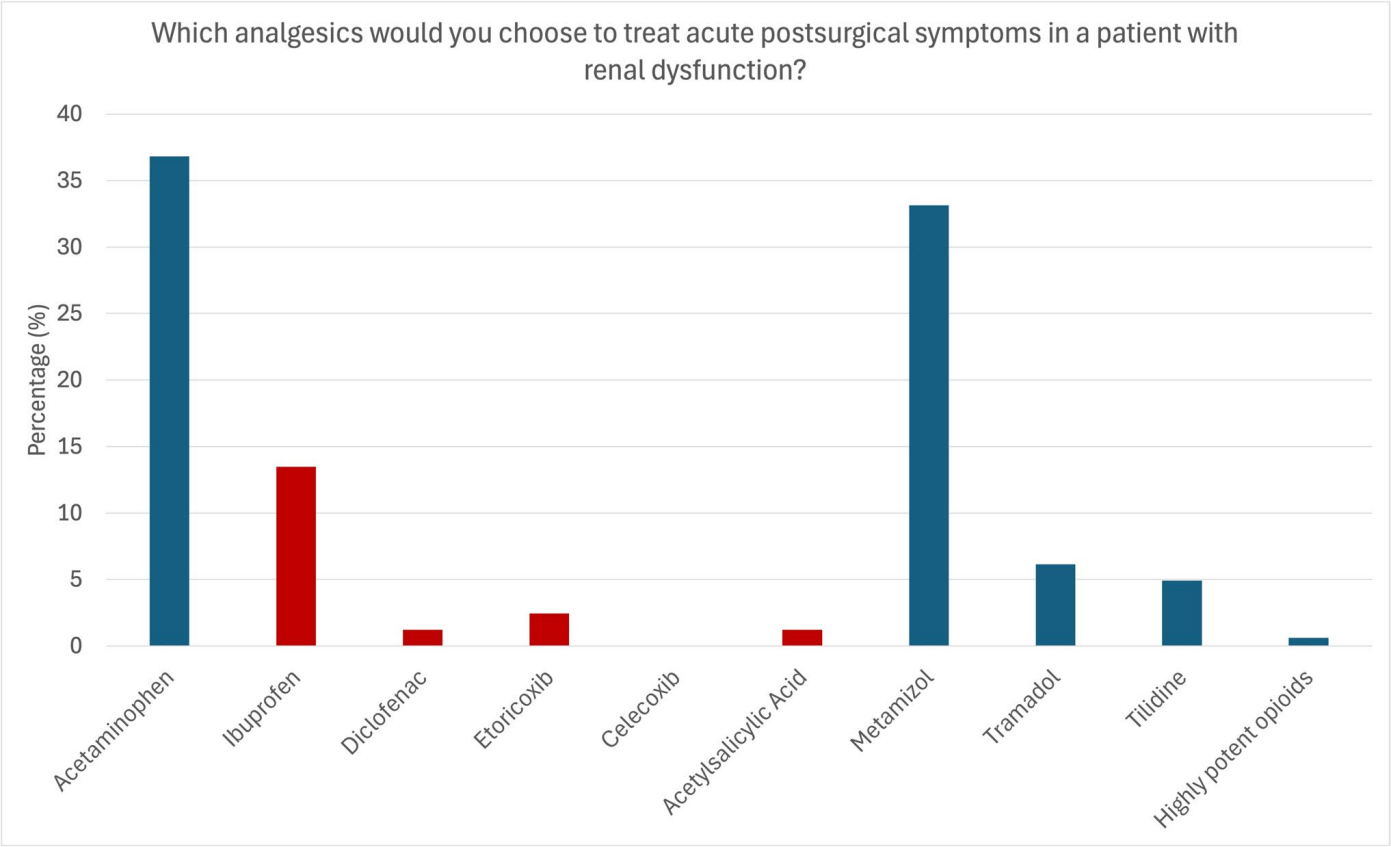
Analgesics preferably prescribed to treat acute pain in patients with renal dysfunction. The participants reported to preferably prescribe/recommend acetaminophen (36.8%) and metamizole (33.1%). Contraindicated analgesics were used in 18.4% (ibuprofen: 13.5%, diclofenac: 1.2%, etoricoxib: 2.5%, celecoxib: 0%, acetylsalicylic acid: 1.2) and highly potent opioids in 0.6%. Red: Contraindicated medication

The participants were further asked which analgesics they prescribe/recommend for treating acute postsurgical symptoms (e.g., after wisdom tooth removal) in a patient with renal diseases. Most participants (37.18%) indicated they would use acetaminophen, followed by metamizole (33.33%). Ibuprofen was reportedly prescribed/recommended in 13.46% of cases, and low-potent opioids like tramadol and tilidine in 10.9%. Contraindicated medications were reportedly prescribed/recommended in 35.9% of cases (ibuprofen, diclofenac, selective COX-2 inhibitors, acetylsalicylic acid) (Fig. 7).

**Fig. 8:**
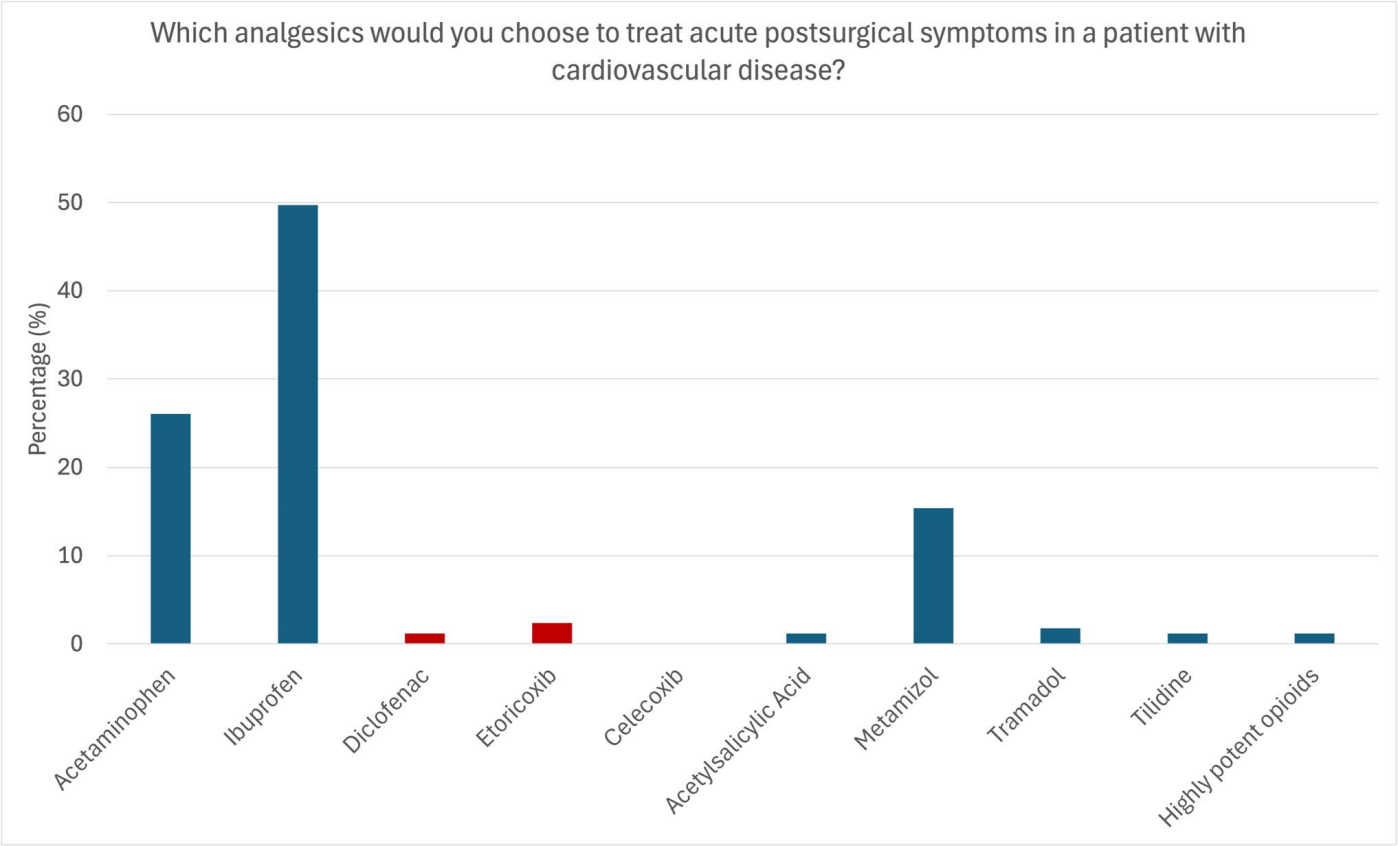
Analgesics preferably prescribed to treat acute pain in patients with cardiovascular disease. The participants reported to preferably prescribe/recommend ibuprofen (49.7%), acetaminophen (26%), and metamizole (15.4%). Contraindicated analgesics were used in only 3.6% (diclofenac: 1.2%, etoricoxib: 2.4%, celecoxib: 0%) and highly potent opioids in 1.2%. Red: Contraindicated medication

Finally, the participants were asked to state which analgesics they prescribe/recommend for treating acute postsurgical symptoms (e.g., after wisdom tooth removal) in a patient with cardiovascular diseases. Half of the respondents reported choosing ibuprofen (50.93%), followed by acetaminophen (26.09%), and metamizole (14.90%). Fewer than 8.1% would use analgesics other than those. Contraindicated medications were used in 3.12% (diclofenac, selective COX-2 inhibitors) (Fig. 8).

## Discussion

Postoperative dental pain typically peaks within the first six hours and persists for up to three days, underscoring the importance of short-term and preemptive analgesia [5].

In 2018, the American Society of Anesthesiologists and associated organizations recommend individualized analgesic regimens based on medical history to minimize adverse outcomes [20]. In our survey, however, adjustment of prescriptions based on patient risk factors was rarely reported. Only 17.14% of participants mentioned allergies as influencing their choice, followed by renal (10.86%) and liver diseases (8.29%).

Current international guidelines, including the 2024 American Dental Association Science and Research Institute (ADA) recommendations and German guidelines, emphasize NSAIDs as the first-line therapy for acute dental pain, often in combination with acetaminophen. If pain is not adequately controlled, an opioid may be added to the first-line treatment at the lowest effective dose for short-term usage [21]. This aligns with Cochrane data showing superior efficacy of ibuprofen and ibuprofen-acetaminophen combinations compared to acetaminophen or opioids alone [22–27]. Reflecting national and international recommendations, the participants in this study most commonly prescribed ibuprofen as the first-line treatment for dental pain (72.84%), followed by acetaminophen (28.02%). International prescribing patterns vary, with ibuprofen favored in the U.S. [28], paracetamol and diclofenac in China [29], and mefenamic acid in Iraq [30]. Similarly, Shukla et al. reported NSAIDs as the preferred choice for overall dental pain in the Indian population [27]. Socioeconomic factors influence opioid prescribing, particularly in the U.S [31], despite updated Centers for Disease Control and Prevention (CDC) guidelines promoting NSAIDs as first-line therapy and advocating minimal opioid use [32].

The ADA’s evidence-based clinical practice guideline for the pharmacologic management of acute dental pain recommends nonpharmacologic and nonopioid therapy as a first-line approach for acute pain lasting less than a month. NSAIDs are emphasized as the primary treatment option, with evidence suggesting that opioids can cause pain exacerbation and might increase the need for rescue medications in patients with acute postsurgical pain. The superiority of NSAIDs is attributed to their anti-inflammatory effects and their ability to inhibit peripheral sensitization. According to the German guidelines on acute postsurgical pain [33], NSAIDs should be preferred over acetaminophen due to their more substantial impact [32].

Metamizole is a prescription-only analgesic in Germany. The number of metamizole prescriptions in Germany has steadily increased in recent years, accompanied by a rise in reports of potentially severe adverse drug reactions. Therefore, the Federal Institute for Drugs and Medical Devices (BfArM) highlighted the importance of appropriate indication and adherence to precautions and warnings [34]. A potential adverse effect of metamizole treatment is the development of agranulocytosis, which can be fatal [34]. According to the data collected during this study, metamizole was prescribed for treating dental pain in 19.49%, reflecting the aforementioned increase in prescriptions. However, it may be anticipated that some of the prescriptions might not adhere to the strict criteria set by the BfArM and, therefore, are contraindicated.

Moore and colleagues summarized the current evidence in dental pain management and proposed a pain-adapted therapeutic regimen. For patients with mild pain severity, they recommended ibuprofen 200–400 mg every 4–6 h as needed. For mild to moderate pain, a single dose of analgesia with ibuprofen 400–600 mg at fixed intervals over 24 h was advised. In cases of moderate to severe pain, the combination of ibuprofen 400–600 mg and acetaminophen 500 mg was suggested at fixed intervals over 24 h [25]. 87.36% of participants recommended ibuprofen post-wisdom tooth surgery, mostly at 600 mg three times daily. Alarmingly, some prescriptions exceeded recommended maximum daily doses, and only a minority used combination therapies as currently advised.

For chronic pain, ibuprofen and diclofenac were most frequently prescribed, whereas adjuvant therapies such as antidepressants were used only rarely. Treatment duration was reported to be relatively short, with 40.60% of participants indicating a treatment period of 4–7 days. According to a recent review by Kämmerer et al., utilizing topical anaesthesia, capsaicin, and systemic medications (tricyclic antidepressants, antipsychotics, and serotonin-norepinephrine-reuptake inhibitors) was significantly more effective for neuropathic pain than nonopioid analgesics [35]. The CDC recommends NSAIDs for chronic orofacial pain only when an inflammatory component, such as osteoarthritis or arthritis of the temporomandibular joint, is present. Due to risks like hypertension, kidney insufficiency, and heart failure, NSAIDs should be used at the lowest effective dose and for the shortest duration. Acetaminophen is not recommended, as it has shown no benefit for chronic orofacial pain. Instead, the CDC advocates using adjuvant analgesics such as anticonvulsants and antidepressants, particularly for neuropathic pain conditions, and emphasizes the importance of interdisciplinary care [32].

Preemptive analgesia aims to achieve therapeutic analgesic blood levels before surgery, thereby reducing inflammation and postoperative pain. A systematic review by Centira Filho et al. showed that preoperative NSAID administration significantly decreased postoperative pain and analgesic consumption [36]. A 2024 study found that 90 mg of etoricoxib given 30 min before tooth extraction reduced pain by three points on a visual analog scale for up to 24 h [37]. Mattos-Pereira et al. compared different analgesics for implant surgery and reported the lowest pain scores with etoricoxib, while acetaminophen was less effective [38]. In our study, only one-third of dentists reported using preemptive analgesia, predominantly prescribing ibuprofen. Despite extensive research since 1991, its implementation in dental practice remains limited, possibly reflecting educational gaps [39].

Management of dental pain in medically compromised patients revealed frequent prescription of contraindicated medications, especially in patients with liver (27.45%) and renal disease (18.4%). Correspondingly, in their cross-sectional survey, Mahdi et al. observed 15.3% of respondents to consider a cautious prescription of NSAID only in patients with a history of peptic ulcer and gastrointestinal bleeding, but neglecting other systemic side effects [30]. In their population-based study, Ingrasciotta et al. reported on inappropriate ketorolac/indomethacin use in 9.2% of elderly patients. Furthermore, at least half of the patients with chronic kidney disease or congestive heart failure were prescribed NSAIDs, while these drugs should be avoided [40]. These data indicate a low awareness of the medication-specific contraindications of analgesics, which is even more concerning given that, according to recent studies, the prescription rates of ibuprofen in Germany have risen from 60.4% to 79% in 2021. At the same time, the number of chronically ill and elderly patients is increasing, making serious side effects more likely [41].

Interestingly, metamizole was prescribed more frequently in patients with risk factors, increasing from 13.15% in healthy individuals to 25% in patients with liver disease and 33.12% in those with renal insufficiency. This trend is concerning given metamizole’s association with severe liver failure and its limited safety data for long-term use in patients with hepatic or renal impairment [42, 43]. Additionally, co-medication with metamizole and acetylsalicylic acid has been linked to increased mortality after myocardial infarction, suggesting a need for caution [44].

Moreover, opioid prescriptions were notably higher among patients with risk factors (liver disease: 10.6% weak opioids, 4.4% strong opioids; renal disease: 11% weak opioids), whereas they were almost absent in healthy individuals. To our knowledge, no previous study has assessed the frequency of prescribing contraindicated analgesics in dental practice, underscoring the necessity for further research.

Out of 1,746 contacted private practice dentists in Rhineland-Palatinate, 232 completed the questionnaire, resulting in a response rate of 13.29%. Factors contributing to the limited engagement may include survey fatigue, time constraints in clinical practice, and a perceived lack of relevance. The anonymity of the questionnaire, although ensuring privacy, may have reduced participants’motivation. The generally low engagement mirrors the limited focus on analgesia in clinical practice.

Due to the anonymized data collection, it was not possible to analyze prescription and recommendation patterns according to specific dental specialties. Although participants from various specialties were included, subgroup analyses could not be performed, which should be considered when interpreting the results. Furthermore, the use of open-ended questions may have limited the systematic capture of influencing factors. The terminology used in the questionnaire (e.g.,’pain medications’) could have caused minor ambiguity regarding adjuvant therapies. However, given the participants’ professional background, a correct interpretation is assumed.

This survey focused primarily on single-agent prescriptions to maintain clarity and optimize participation. Although combined therapies could be indicated via multiple-choice and free-text responses, a structured evaluation of combination treatments was not the primary aim. Future studies should specifically address prescription patterns involving combined analgesic regimens.

## Conclusion

The range of analgesics prescribed/recommended by dentists remains limited and largely independent of the specific indication. According to current guidelines, NSAIDs, particularly ibuprofen, are the most commonly used analgesics. However, many contraindicated analgesics are reportedly prescribed/recommended for patients with certain risk factors. The long-term use of COX-2 inhibitors, approved only for short-term use in dentistry, also highlights a need for further education. Preemptive analgesia, an essential tool in pain prevention, is utilized by only one-third of the participants, indicating a need for greater awareness and use of this approach.

## Data Availability

No datasets were generated or analysed during the current study.
